# Inclusión laboral y medicalización: neurolépticos como actantes en las experiencias de personas trabajadoras diagnosticadas de esquizofrenia en Chile

**DOI:** 10.18294/sc.2026.5903

**Published:** 2026-03-10

**Authors:** Ernesto Bouey Vargas, María José Reyes Andreani

**Affiliations:** 1 Doctor en Ciencias Sociales. Profesor, Facultad de Psicología, Universidad Diego Portales, Santiago, Chile. ernesto.bouey@mail.udp.cl Universidad Diego Portales Facultad de Psicología Universidad Diego Portales Santiago Chile ernesto.bouey@mail.udp.cl; 2 Doctora en Psicología Social. Académica, Departamento de Psicología, Universidad de Chile, Santiago, Chile. mjrandreani@u.uchile.cl Universidad de Chile Departamento de Psicología Universidad de Chile Santiago Chile mjrandreani@u.uchile.cl

**Keywords:** Esquizofrenia, Agentes Antipsicóticos, Condiciones de Trabajo, Estigma Social, Chile

## Abstract

Las personas diagnosticadas de esquizofrenia presentan bajas tasas de participación laboral, fenómeno que ha sido abordado, de forma predominante, desde enfoques biomédicos que asocian inclusión laboral con estabilización farmacológica. Sin embargo, existe escasa investigación que analice cómo el uso cotidiano de neurolépticos incide en las experiencias laborales de quienes trabajan con este diagnóstico. El objetivo de este estudio fue comprender el papel de los neurolépticos -concebidos como actantes- en las experiencias de personas trabajadoras diagnosticadas de esquizofrenia en Chile y sus implicancias para la inclusión laboral. Se desarrolló una investigación cualitativa etnográfica entre 2022 y 2024 en la Región Metropolitana de Chile, que incluyó observación participante en diversos escenarios de la vida cotidiana y laboral, y 86 entrevistas (53 a personas trabajadoras diagnosticadas y 33 a personas de su entorno). El análisis, inspirado en la teoría del actor-red y en la noción de enactment, permitió identificar que el diagnóstico y el neuroléptico se ensamblan como una práctica para toda la vida, configurando una experiencia marcada por tensiones. Si bien los fármacos son valorados por aliviar la angustia, su ensamblaje al diagnóstico y sus efectos secundarios -sedación, alte­raciones motoras, salivación, aumento de peso, aplanamiento afectivo- median en el cuerpo, el psiquismo y la vida social, dificultando el desempeño laboral y exponiendo a las personas a estigma y discriminación.

## Introducción

Las personas diagnosticadas de esquizofrenia constituyen uno de los grupos sociales más excluidos a nivel global. Diversas investigaciones han evidenciado que sus tasas de participación laboral son extremadamente bajas, con estimaciones que oscilan entre un 4% y un 10%, pero que pueden alcanzar el 50% dentro de programas específicos de empleo en países del norte global^(1,2^. En Chile, si bien no existen datos que permitan establecer su tasa de empleabilidad, al revisar bases de datos públicas se puede suponer tasas de empleo entre el 19% y el 3%^3^^,^^4^, dependiendo de la metodología de medición. Esta exclusión cobra aún más relevancia si se considera que el trabajo ha sido identificado como un elemento fundamental para la recuperación, mejora del bienestar, autoestima e inclusión social de quienes han recibido este diagnóstico^5^^,^^6^^,^^7^.

El campo de investigación sobre esquizofrenia y empleo ha estado históricamente dominado por enfoques biomédicos y psicopatológicos. Desde estas perspectivas, el acceso al trabajo ha sido conceptualizado como el resultado de la estabilización clínica, alcanzada principalmente mediante tratamientos farmacológicos prolongados^8^^,^^9^^,^^10^. En esa línea, se ha buscado identificar aquellos fármacos que facilitarían con mayor efectividad el ingreso o la permanencia laboral^11^. Esta mirada ha sido reforzada por el desarrollo de programas como el *Supported Employment* o el *Individual Placement and Support*, en los que la adherencia al tratamiento farmacológico es central para la inclusión laboral^12^^,^^13^.

No obstante, diversas investigaciones han advertido que esta perspectiva resulta limitada para comprender los procesos de inclusión laboral. En particular, se ha señalado, desde hace más de dos décadas, que la baja empleabilidad no puede explicarse únicamente por el cuadro clínico y su tratamiento, sino que debe considerarse el entrecruzamiento de factores sociales, económicos, contextuales, familiares y educativos^2^^,^^14^. Al respecto, el estigma ha sido identificado como uno de los principales obstáculos, afectando tanto la posibilidad de obtener como de mantener un empleo^15^^,^^16^^,^^17^, enfatizando que más que el cuadro clínico y sus manifestaciones psicopatológicas, la inclusión laboral está mediada por los contextos sociales.

Un aspecto que ha sido menos explorado es el papel que juegan los tratamientos farmacológicos en estos procesos. Históricamente, su uso masivo para el tratamiento de la esquizofrenia se sitúa en las décadas de 1950-1960, en el contexto de la “revolución farmacológica” con el surgimiento de la clorpromazina, cuyo principal efecto era la sedación y pérdida de interés por el entorno y por sí mismos^18^. Se crea el término *neuroléptico*, pues se asociaba la eficacia terapéutica a los efectos neurológicos observables en espasmos musculares, inquietud, temblores, entre otros. Luego surgen tratamientos llamados de segunda generación con compuestos como la clozapina. Con el tiempo el concepto de *neuroléptico* fue reemplazado por el de *antipsicótico*, reflejando un cambio hacia un modelo centrado en la enfermedad, en el que se asumía que los medicamentos actuaban sobre los procesos biológicos subyacentes de las enfermedades mentales, como la esquizofrenia, corrigiendo desequilibrios químicos en el cerebro. Esta nueva concepción invisibilizó en la clínica y la investigación la relevancia de los efectos neurológicos^19^. En tal sentido, en esta investigación, utilizaremos preferentemente el término neuroléptico porque un foco importante de análisis son las experiencias ligadas a los efectos secundarios.

Al respecto, se ha mostrado que los antipsicóticos de segunda generación presentan efectos secundarios que inciden negativamente en la vida social y funcional de las personas^20^^,^^21^. Entre estos se destacan la sedación, disfunción cognitiva, aumento de peso, disfunción sexual, embotamiento emocional, deterioro de la capacidad de concentración, temblores, alteraciones en la motricidad^22^^,^^23^^,^^24^^,^^25^^,^^26^^,^^27^^,^^28^, problemas metabólicos que pueden llevar a diferentes déficit cognitivos^29^, aumento de peso, hiperglucemia y dislipidemia^30^, aumento de la probabilidad de sufrir diabetes^31^.

Desde estudios cualitativos y etnográficos, se ha observado que los neurolépticos no solo impactan en lo físico y funcional, sino también en la construcción de la identidad de las personas, en su autopercepción y en su manera de relacionarse con el entorno. Estroff^32^, en una investigación pionera, mostró cómo los efectos de los fármacos influían en la forma en que los sujetos se concebían a sí mismos como “enfermos mentales”, reforzando procesos de estigmatización e internalización del diagnóstico. Investigaciones posteriores han reforzado esta idea al señalar que los tratamientos farmacológicos prolongados pueden producir una vivencia de desvitalización, aplanamiento afectivo y pérdida del sentido de agencia personal^25^^,^^26^^,^^33^.

Adicionalmente, hoy en día se ha comenzado a cuestionar la idea de que los tratamientos farmacológicos deban ser indefinidos. Investigaciones longitudinales muestran que, luego de los primeros años posteriores al diagnóstico, muchas personas pueden reducir o suspender los neurolépticos sin que ello implique un peor pronóstico e incluso alcanzando mejores niveles de recuperación funcional^28^^,^^34^. No obstante, quienes intentan descontinuar o reducir la medicación enfrentan múltiples barreras, desde el temor a la recaída infundido por profesionales, hasta la ausencia de apoyos institucionales para transitar este proceso^21^^,^^35^.

Esto ocurre tanto en el ámbito médico como en los procesos de intermediación laboral, en los que se solicita, por ejemplo, certificados de “estabilidad clínica” o uso de psicofármacos como requisito para acceder a un trabajo, reproduciendo una lógica de vigilancia médica sobre la vida laboral de estas personas^27^^,^^36^.

Estos antecedentes dan cuenta de una tensión poco explorada entre el uso de neurolépticos y los procesos de acceso al empleo. Aunque existe literatura crítica sobre los efectos adversos de los psicofármacos y sobre las vivencias subjetivas asociadas a su uso^37^^,^^38^, no se han desarrollado estudios que aborden en profundidad las experiencias de las personas trabajadoras diagnosticadas de esquizofrenia con el uso de neurolépticos, y cómo estas vivencias se articulan con las posibilidades y obstáculos para su inclusión laboral.

Conceptualmente, analizaremos el papel de los neurolépticos en tanto agentes en la vida social de estas personas. Siguiendo a Latour^39^, los elementos no humanos también poseen capacidad de agencia (actantes), pues, al insertarse en una red, se ensamblan y modifican el curso de la acción. Por tal razón, se analizará el lugar de los neurolépticos desde las experiencias de las personas diagnosticadas de esquizofrenia.

Annemarie Mol^40^^,^^41^, a través del concepto de *enactment*, postula que diferentes elementos se pueden ensamblar entre sí, como objetos, cuerpos, enfermedad, etc., produciéndose y adquiriendo realidad dentro de prácticas específicas. Esto hace que sean múltiples según las actividades en las que participan. De este modo, los neurolépticos pueden ser cosas distintas en redes de práctica diferentes^42^. Para la autora^41^, los cuerpos no son un todo cerrado, sino que tienen fronteras semipermeables, incorporan objetos externos para funcionar. Por ejemplo, la comida (como una manzana) pasa de ser exterior a ser parte de la estabilidad interna, así como los fármacos. La autora habla de un *cuerpo metabólico* cuya actividad paradigmática es metabolizar (comer, respirar, excretar), enfatizando que sus fronteras son fluidas y la importancia del intercambio constante con el entorno, especialmente, en este caso, con los fármacos. Esta conceptualización permite comprender que los cuerpos son un conjunto de tensiones que deben ser manejadas.

Como muestran diversas investigaciones^43^^,^^44^^,^^45^^,^^46^, las personas trabajadoras diagnosticadas de esquizofrenia deben lidiar con el estigma cotidianamente, y los neurolépticos tienen un rol dentro de estas prácticas. Siguiendo a Goffman^47^, el estigma se define como un proceso relacional en el cual un individuo es identificado con un atributo arbitrario que lo diferencia de los demás, convirtiéndolo en una persona desacreditada o potencialmente desacreditable y que lo lleva a ser discriminado. El estigma tiene consecuencias en la identidad de estas personas, quienes pueden identificarse con ese estigma, revelarse a él, esconder la marca o buscar apoyo en otros. Respecto del estigma asociado a la esquizofrenia (Goffman habla de *defecto del carácter*), al no ser inmediatamente perceptible, la persona podría ocultar y gestionar esa marca para evitar la desacreditación. En este marco, el estigma no es inherente a la persona, sino que se trata de una marca social que se impone a los sujetos. Por tanto, el diagnóstico mismo de esquizofrenia es parte del estigma, una marca que lleva a la desacreditación^48^^,^^49^.

Desde estos antecedentes y concepciones conceptuales, esta investigación tiene como objetivo profundizar en las experiencias con el uso de neurolépticos -en tanto actantes- de personas trabajadoras diagnosticadas de esquizofrenia y sus implicancias para la inclusión laboral.

## Metodología

Esta investigación se desarrolló desde un enfoque cualitativo de tipo etnográfico, orientado a comprender las experiencias vividas de personas trabajadoras diagnosticadas de esquizofrenia, considerando sus significados y prácticas cotidianas^50^^,^^51^^,^^52^. Se siguió una lógica inductiva^53^, situando los relatos en sus marcos interpretativos y contextuales^54^.

Para la selección de las personas participantes, se utilizaron criterios de inclusión territoriales (Región Metropolitana de Chile), clínicos (diagnóstico de esquizofrenia) y laborales (trabajo formal en los dos años previos). La selección combinó muestreo teórico^55^^,^^56^ y por conveniencia, apoyándose también en la técnica de bola de nieve^54^. Se accedió a las personas a través de organizaciones de colocación laboral, de profesionales de salud mental y conocidos del investigador principal, quienes explicaban las características del estudio y entregaban el contacto del investigador para participar de forma voluntaria en la investigación. La selección quedó conformada por 16 personas diagnosticadas de esquizofrenia con experiencia de trabajo formal reciente (Tabla 1), además de 30 personas de su entorno próximo: familiares, compañeros y compañeras laborales, jefaturas directas, profesionales de salud mental y personas trabajadoras de programas de inclusión laboral.

**Tabla 1:** Características de las personas trabajadoras diagnosticadas de esquizofrenia, que participaron del estudio. Chile, 2022-2024.

Pseudónimo	Edad	Género	Nivel educacional	Neuroléptico principal	Pensión de invalidez
Francisco	45	Masculino	Superior incompleta (técnica)	Clozapina	Sí
Pablo	52	Masculino	Superior incompleta (universitaria)	Clozapina	Sí
Cristián	33	Masculino	Media	Clozapina	Sí
Enrique	34	Masculino	Superior (técnica)	Clozapina	Sí
Pedro	57	Masculino	Básica	Risperidona	Sí
Martín	41	Masculino	Superior (universitaria)	Aripiprazol	No
Irene	45	Femenino	Básica	Clozapina	Sí
Osvaldo	39	Masculino	Superior incompleta (universitaria)	Olanzapina	Sí
Marisol	27	Femenino	Básica incompleta	Quetiapina	Sí
Rocío	51	Femenino	Superior (universitaria)	Clozapina	No
Diego	43	Masculino	Básica	Quetiapina	Sí
Pamela	33	Femenino	Media incompleta	Clozapina	Sí
Lucas	34	Masculino	Media	Clozapina	Sí
Nicolás	40	Masculino	Media incompleta	Clozapina	Sí
Rodolfo	53	Masculino	Media incompleta	Clozapina	Sí
Sonia	59	Femenino	Media	Aripiprazol	Sí

Fuente: Elaboración propia. Nota: La educación chilena se estructura de la siguiente manera: Educación parvularia, básica (1° a 8° básico), Media (1° a 4° medio) y Superior (universidades, institutos profesionales y centros de formación técnica).

Las técnicas empleadas fueron observación participante y entrevistas etnográficas. La observación se realizó en diversos escenarios definidos por las actividades cotidianas de las personas participantes, incorporando la movilidad y la multiplicidad de espacios^57^^,^^58^. En tal sentido, se acompañó a las personas en sus actividades cotidianas en la medida que quisieran ser acompañadas. Los escenarios consistieron en controles médicos, traslados varios, hogares protegidos, lugares de búsqueda de empleo como la Oficina Municipal de Información Laboral (OMIL) o fundaciones, servicios públicos, espacios de trabajo, entre otros.

Entre 2022 y 2024, se registraron 32 informes detallados de campo, que integraron descripciones densas, interpretaciones de los sujetos, reflexiones teóricas y elementos metodológicos^59^^,^^60^. Las entrevistas etnográficas, de carácter no directivo, buscaron favorecer la reflexividad y el despliegue subjetivo de las personas entrevistadas^51^^,^^61^. Se grabaron y transcribieron 86 entrevistas: 53 a personas trabajadoras diagnosticadas y 33 a personas de su entorno. Estas entrevistas se realizaron durante las observaciones participantes, de modo que se llevaron a cabo en los diversos espacios descritos anteriormente. En su mayoría se trató de entrevistas individuales, pero también hubo algunas colectivas. La participación fue voluntaria y se les aseguró confidencialidad.

El análisis fue simultáneo a la producción de datos, bajo una lógica recursiva coherente con la etnografía y la teoría fundamentada de perspectiva construccionista^53^^,^^62^. Se utilizó el software NVivo para organizar la información. La codificación fue inicialmente abierta y los investigadores e investigadoras fueron construyendo códigos inductivos, relacionándolos entre sí y creando categorías. En este proceso se construyeron hipótesis que luego fueron comprobadas o desestimadas al analizar los datos y en el trabajo de campo junto a los participantes (muestreo teórico). Al ir consolidando las categorías y sus relaciones mediante la categorización axial, se continuó construyendo hipótesis y comprobándolas con las personas participantes hasta alcanzar la saturación teórica. Finalmente, se construyó una codificación selectiva para estructurar los hallazgos respecto de la experiencia con los neurolépticos en torno al acceso al empleo.

El estudio contó con la aprobación del Comité de Ética de la Investigación de la Facultad de Ciencias Sociales, Universidad de Chile (Informe N° 25-27/2022), institución en la que se alojó la investigación. Todas las personas participantes fueron informadas de las características de la investigación y firmaron un consentimiento por escrito donde se garantizaba la confidencialidad, voluntariedad y resguardo de la identidad de las personas involucradas mediante la utilización de pseudónimos y anonimización de otras personas o lugares.

## Resultados

### Ensamblaje entre diagnóstico de esquizofrenia y neurolépticos para toda la vida

Un aspecto que marca las vidas de las personas diagnosticadas de esquizofrenia es que se les ha transmitido que el diagnóstico es para toda la vida. Esto quiere decir que nunca les darán el alta médica ni les dirán que se han curado/sanado, lo que conlleva a que deberán mantener la medicación hasta su muerte. Nicolás afirma:

“*Yo tengo claramente que esto no tiene cura por el momento, no existe la cura para la esquizofrenia, está más que claro. Y tengo que seguir tomándome los medicamentos, ser responsable, porque tengo más que claro que si no me tomo los medicamentos va a llegar un momento en que mi cuerpo lo va a necesitar, mi cerebro también y voy a descompensarme, voy a caer hospitalizado*”. (Nicolás, entrevista del 17-07-2023)

Los mismo refiere Enrique:

Investigador: “*Me decías que el diagnóstico de esquizofrenia dura toda la vida, que no te pueden dar de alta”.*

Enrique: “*Sí. Era porque hay que seguir con los medicamentos siempre…”* (Enrique, entrevista del 24-01-2023)

Todas las personas trabajadoras diagnosticadas de esquizofrenia que participaron de esta investigación mantenían sus tratamientos farmacológicos. Asimismo, las y los profesionales de la salud mental con los que se tuvo contacto sostuvieron que el tratamiento farmacológico es fundamental para el tratamiento de la esquizofrenia, argumentando que, si los pacientes lo descontinúan, vuelven a presentar crisis psicóticas.

El diagnóstico crea una nueva práctica cotidiana que consiste en ingerir el fármaco tres veces al día, en la mañana, al medio día y al anochecer. En algunos casos se administran los llamados *neurolépticos de depósito* que se inyectan una vez al mes en pacientes con dificultad de adherencia a tratamiento. Pero todas las personas participantes de esta investigación mantenían la práctica de ingerirlos tres veces al día. De esta forma, diagnóstico y neurolépticos se transforman en un ensamblaje para toda la vida de las personas diagnosticadas de esquizofrenia, el que se mantiene diariamente por indicación médica. 

### Tensiones con el uso de psicofármacos

Aunque las personas participantes de esta investigación mantenían sus controles psiquiátricos y sus medicaciones, muchos muestran un marcado conflicto con su uso. Por un lado, están quienes refieren que se encuentran bien con sus tratamientos actuales, que ayuda a tener menos ansiedad y angustia. En general, agradecen porque señalan a los medicamentos como responsables de estar mejor y algunos esperan que continúen ayudándolos como lo han hecho hasta ahora. Por ejemplo, Pablo menciona:

“*Con este medicamento ando bien relajado, me permite ser constante en lo que hago, en un trabajo me puedo mantener constante o, si estudio, me va a hacer mantenerme constante y levantarme temprano a trabajar, no se me hace muy pesado*”. (Pablo, entrevista del 25-05-2023)

Por otro lado, otras personas desean dejar los neurolépticos porque se encuentran bien y afirman que no los necesitan, tanto porque desean evitar los efectos secundarios que conllevan como porque dejar de tomarlos les reafirmaría que están sanos y no necesitan tratamientos. Osvaldo dice:

“*Me encantaría tomar menos pastillas y sentirme bien, sano, sí, pero, si no se puede, da lo mismo*. […] *Sí, sí, no es algo que puedas hacer como mucho tomando los medicamentos, son efectos secundarios no más, hay que ver el mal menor no más o algo así”*. (Osvaldo, entrevista del 13-7-2023)

“*Pedro, por otro lado, insistió en varias oportunidades que deseaba dejar de tomar sus medicamentos porque estaba bien, pero que los médicos le insistían que no debía descontinuar el tratamiento*”. (Observación del 5-1-2023).

Ocurre, de este modo, que el ensamblaje del diagnóstico y el neuroléptico es vivido por los participantes como una tensión, pues les ha hecho sentirse bien respecto de la ansiedad y angustia, pero produce sufrimiento a causa de los efectos que conlleva y la significación de ser enfermos mentales.

### Neuroléptico como mediador entre cuerpo, psiquismo y vida social

El *ensamblaje* diagnóstico/neuroléptico opera como *mediador* (Latour, 2005) de las experiencias de las personas diagnosticadas de esquizofrenia en tanto las transforma de manera significativa. 

Es común escuchar de personas que suben de peso por los medicamentos psiquiátricos. Por ejemplo, Osvaldo (entrevista del 13-7-2023) cuenta que ahora está bien de peso, pero que llegó a pesar 93 kilos a causa de los medicamentos. Por lo general, los problemas derivados de los antipsicóticos afectan directamente en su vida diaria. Así Cristián afirma que los medicamentos le producen problemas estomacales:

“*Con la clozapina fue el tema de la guata* [estómago], *porque soy muy sensible de la guata. Yo te como cualquier cosa y me hace mal.* […]. *Yo antes no tomaba la pastilla y no era así. Podía comer queso o podía tomar leche. Ahora ya me hace mal la leche, los lácteos, todo eso*”. (Cristián, entrevista del 4-5-2023)

También se reportan otros signos en los cuerpos de estas personas. En una visita a un hogar protegido, una de las cuidadoras comenta:

“*La clozapina, por ejemplo, te provoca taquicardia. Yo no sé si te diste cuenta que* [nombre de un residente] *tiene un color como azul violáceo en la piel. Porque anda por aquí. Él tiene un tema cardiaco. Le han bajado la clozapina porque le provoca taquicardias. También les provoca estitiquez.* [Nombre de otro residente], *por ejemplo, sufre toda la semana para ir al baño por el medicamento. Hay otros medicamentos que los mantienen así, tiritando*. […] *Y es un impedimento, claramente para salir*”. (Cuidadora, entrevista del 24-8-2023)

Pablo cuenta que tuvo distonía (contracciones musculares involuntarias) por un medicamento que recibió a los 19 años:

“*Lo que causa distonía es el Haldol.* […]. *Yo tenía como diecinueve, veinte años y el doctor me dio Haldol*^®^ *y me daba distonía, pero para contrarrestar eso me daba Tonaril*^®^”. (Pablo, entrevista del 25-5-2023)

Marisol comentó en diversas oportunidades lo angustiante que era para ella cada vez que le ocurría una distonía. En su caso, la manifestación consiste en que los ojos se le mueven hacia arriba y tensionan el músculo. Describe que cuando le ocurre no puede hacer nada más, porque no ve bien. Además, las otras personas que están con ella se asustaban y la rechazan con miedo. Por esa razón, durante algunos meses, no salió de su casa para evitar que le ocurriera estando en la calle y sin ayuda.

Los síntomas motrices no se reducen solo a la distonía. En una observación en un hogar protegido psiquiátrico del 25-5-23 se observa extrema rigidez en los movimientos corporales de muchos residentes.

“...*me llama la atención esa rigidez de cuerpo en algunas personas con diagnóstico de esquizofrenia del hogar. Un hombre pasa al lado mio sin levantar la cabeza, brazos colgando al lado del cuerpo, sin balanceo y una expresión facial entre ido y sorprendido*.” (Observación del 25-5-23, en hogar protegido)

Caminan sin mover las manos, algunos con la cabeza gacha y con movimientos mecánicos:

“*Pasa un hombre de unos 30 años acompañado de 2 funcionarios, un hombre y una mujer probablemente técnicos en enfermería o enfermeros. Lo llevan tomado de un brazo, se ve muy rígido en sus movimientos, mirada gacha, como si no pudiese doblar las rodillas ni mover los brazos con libertad. Marisol se queda mirando y, cuando se va, me dice que le da mucha pena ver eso, que cuando estuvo hospitalizada así quedaba la gente por los fármacos y el electroshock, que ella también estuvo así*.” (Observación del 20-10-22, con Marisol, en hospital psiquiátrico).

Esos mismos tipos de movimiento son los que uno puede observar dentro de los hospitales psiquiátricos (Observación del 25-1-23 con Enrique; del 4-5-23 con Cristian; del 14-7-23 con Irene).

Las personas participantes de esta investigación señalan que los efectos que han padecido de los neurolépticos, especialmente cuando se presentan de forma aguda o son más manifiestos, las obligan a aislarse y evitar el contacto con otros, incluido trabajar o buscar trabajos. Refieren que los medicamentos para la esquizofrenia representan una importante barrera para socializar con otros, lo que las lleva al aislamiento. En tal sentido, Osvaldo refiere que los fármacos le han provocado temblores en las manos, tan marcados que le afecta su vida social:

[Los fármacos] “*Tienen efectos secundarios. Yo no sé si es porque tomo muchas pastillas, pero tengo muchos temblores en las manos siempre y eso es súper incómodo. De hecho, conocer gente que le gustan los juegos de mesa para mí es como un no, no soy capaz de mover bien una pieza a otra sin que se note que estoy tiritando y que la gente vea eso y no cache porqué me complica ¿cachai? Como lo social.* […] *Justo ahora estoy hablando con una persona que es diseñador gráfico y me está incentivando a dibujar, cosa que yo amaba dibujar y pintar, pero por lo del temblor lo dejé*”. (Osvaldo, entrevista del 13-7-2023)

La salivación es muy relevante para estas personas. Diversas personas participantes salivan o se les seca la boca a causa de los fármacos psiquiátricos. Algunos refieren que deben hacer un esfuerzo adicional para que la saliva no se les caiga por la boca. Otros deben disponer de una botella de agua constantemente para la sequedad. Enrique cuenta: “*Cuando empecé con los medicamentos, la saliva… la risperidona me dejaba tieso. La risperidona me dejaba tieso… la saliva*.” (Enrique, entrevista 25-1-2025). Francisco comenta que la saliva es lo que más le preocupa, que durante la noche y en el día saliva en exceso (Observación del 5-5-2023).

Para Pablo y Cristián el problema de la salivación se debe a la clozapina y ocurre principalmente en la noche, mientras duermen. Para muchos otros esto ocurre en el día y no necesariamente al comienzo de los tratamientos, sino que comenzó tardíamente, un año después. Nicolás dice “*la clozapina te tumba, se te cae la baba y toda la cuestión*” (Nicolás, entrevista del 17-7-2023).

Las personas participantes refieren que, a causa de la salivación, evitan conversaciones con otras personas, pues han notado que a otros les provoca rechazo, sin embargo, no lo pueden controlar. Algunos, como Marisol, Nicolás o Cristián, se han visto obligados a explicar a su interlocutor en contextos laborales, como entrevistas de trabajo o compañeros y jefaturas que es un problema de los medicamentos.

Otro de los efectos secundarios que destacan es la sedación durante el día, lo que incide de gran manera en la búsqueda y obtención de un empleo. Osvaldo dice que el medicamento lo tranquiliza, pero lo mantiene menos alerta, por lo que duda si es mejor o peor.

“*De hecho, cuando empecé a ponerme muy nervioso con la pega me dieron clonazepam que al final me dejaba más tranquilo, pero menos alerta a las cosas que pasaran. Entonces no sabía si me servía para trabajar. No sé si me jugó a favor o en contra, me sentía mejor, pero no estaba funcionando mejor*”. (Osvaldo, entrevista del 13-7-2023)

Martín comenta que sentía un cansancio agotador con los medicamentos anteriores, pero ahora puede trabajar más horas porque la nueva medicación no lo agota tanto.

“*O sea, sí me da un cansancio, pero tendría que estar como…con… muchas horas de falta de sueño y con mucho trabajo para sentirme muy cansado. Pero ya no es ese cansancio agotador que sentía antes*” [con la medicación anterior]. (Martín, entrevista del 22-9-2023)

Irene refiere que los neurolépticos le producen sueño, por lo que siempre tiene que hacer un esfuerzo para mantenerse despierta. Lo mismo dice Pamela, que por esa razón conversó con su psiquiatra y logró que concentrara la mayor dosis en la noche, de modo de tomárselos y acostarse a dormir, aunque aún continúa sintiéndose sedada durante el día. Lucas comenta que se quedaba dormido durante el día realizando diversas actividades y que perdió un trabajo a causa de este efecto secundario: “*la verdad es que a veces me quedaba dormido porque como tomaba mucha pastilla, mucho medicamento me quedaba dormido*” (Lucas, entrevista del 4-1-2023).

Nicolás es muy preciso al indicar que ese efecto del neuroléptico le impedía efectuar actividades importantes de su vida, como realizar cursos de capacitación para encontrar un trabajo:

“*Ahora el tema de la clozapina cuando yo estaba tomando 750 miligramos en la noche, estaba estudiando en* [fundación de inclusión laboral] *la primera vez, cosa que no pude continuar porque andaba muy mal, muy dopado. Llegaba a la clase y estaba así, como así, como quedándome dormido y se me caía la baba, así, a ese nivel. Y la profesora me dijo, sabes Nicolás quiero conversar contigo después cuando termine la clase. Y me dijo ¿qué te pasa Nicolás? Me dijo, estás mal, se te cae la baba, apenas pones atención en la clase, decía. Deben ser los medicamentos que en ese momento yo andaba en otra.* (Nicolás, entrevista del 17-7-2023)

Además de no poder realizar las actividades que desean, la sedación interfiere en las relaciones con otras personas. Varias personas fueron rechazadas en trabajos porque los notaron muy sedados, pero también es algo que provoca reacciones en los cercanos, como las amistades. Algunas personas relatan que sus amistades se burlaban por quedarse dormidas o no poder concentrarse, otras amistades se alejaron, pero también hubo quienes los quisieron ayudar. 

El relato de Pamela es muy significativo al respecto. Ella se había ido a vivir con una amiga fuera de Santiago, estaba muy feliz y se sentía muy independiente, pero su amiga comenzó a compadecerse porque la veía tomar medicamentos que le provocaran sedación:

“…*porque ella me vio dopada. Me veía cuando me tomaba los remedios y quedaba un poquito dopada. Y me decía, que le daba pena verme así*”. (Pamela, entrevista del 4-1-2024)

Otra área en la que las personas reportan que el fármaco tiene agencia, tiene que ver con las emociones. Varias personas participantes afirman que la medicación les ha ayudado a bajar la angustia, pero que también trae como consecuencia un efecto en las emociones que las lleva a experimentar menos interés en asuntos que antes les eran importantes. Martín expresa sus dudas respecto del origen de esa sensación:

“*No me interesa mucho el dinero ni las cosas materiales. Porque… no sé si… eh, le conté, pero… yo soy una persona que tiene las emociones aplanadas. Eh, no me acuerdo bien cómo se llama el síntoma… Pero es que no siento rabia, rencor, odio, alegría, tristeza, ningún tipo de emoción. Es por los medicamentos, me dijeron a mí una vez. Pero no pienso dejar los medicamentos, porque no quiero recaer de nuevo en la enfermedad, porque ya me dijeron que si una tercera vez no saldría del hospital*”. (Martín, entrevista del 22-9-2023)

Esto le ha afectado también en su vida personal, por ejemplo, dice que ya no le importan los problemas, que incluso se le olvidó cómo era su vida antes del diagnóstico de esquizofrenia, pero tampoco le importa recordarla. Afectando incluso el interés por establecer vida de pareja:

Martín: “*Antes tuve* [pareja]… *pero después de la enfermedad no… no pasa nada no más, no, no, no pasa nada nomás, si no hay emociones, menos lo otro*”.

Investigador: “*¿Cómo no pasa nada? ¿No sientes atracción?*”

Martín: “*No, nada, nada, nada*”. (Martín, entrevista del 22-9-2023)

Martín afirma que el desinterés abarca las diferentes esferas de su vida, incluido el trabajo. Refiere que no le interesa trabajar, ni tener dinero, que lo hace solo por no ser una carga para su padre que lo cuida. Reflexiona además que esa podría ser la causa de que muchas personas diagnosticadas de esquizofrenia no trabajen ni les interese hacerlo.

### Gestión cotidiana de los neuroléptico

Nos encontramos entonces con que el ensamblaje diagnóstico/neuroléptico produce tensión en la experiencia de estos sujetos porque, por un lado, se entiende como necesario a causa del diagnóstico de esquizofrenia pero, por otro, se constituye en un actante que modifica el cuerpo, el psiquismo y la vida social, dificultando o imposibilitando realizar la vida con normalidad y trabajar.Esta tensión la expresa muy claramente Sonia:

“*La vida no es fácil tampoco. O sea, con muchas dificultades, porque... esperando que nos acepten, la gente, así como... como zombis. Porque no hay otra palabra como describirlo. Es como uno, como un zombi. Estando... porque hay que dopar* [medicar] *a las personas, porque, si no, llegan al extremo de irse de este mundo nomas. Hay que hacerlo. Y yo no quería irme de este mundo tampoco. Pero, dificultoso, o sea, siempre tratando de decir, no, tú puedes tú lo vas a lograr*”. (Sonia, entrevista del 24-08-2023)

Entonces, valora los medicamentos, pero debe sopesar continuamente cuánto le ayuda el fármaco y cuánto la perjudica. Afirma que le permite estar más tranquila de los pensamientos que la angustian, pero la sedan mucho, por lo que no puede rendir bien en el trabajo.

“*Sí, pues me da mucho sueño. Yo... aparte yo, como conozco a mi cuerpo. La doctora no: ‘que tómesela entera’, una cosa así. Es que ya ni me acuerdo, la cuestión es que yo me tomo la mitad de la mitad de las pastillas. Para no tener tanto sueño, para cumplir también, para ser responsable. Porque yo ya estoy en este barco... ya estoy. Entonces tengo que cumplir con lo que... y ser responsable, más que nada en la vida. Pero yo... yo quiero cumplir. Lo único que quiero es cumplir, seguir adelante. No quiero andar como un zombi tampoco*”. (Sonia, entrevista del 24-08-2023)

Este manejo de los medicamentos de Sonia no es un logro exclusivo de ella. En diferentes conversaciones, las personas participantes relataron momentos en que debieron modificar las indicaciones de los médicos, especialmente para estar más despiertas, tener menos efectos físicos o porque estaban muy angustiadas. 

Comprendiendo que el cuerpo se construye, el uso de fármacos no es una simple adhesión pasiva a una norma, sino una práctica cotidiana donde el individuo gestiona activamente un conjunto de tensiones. Esta gestión implica a menudo intervenir de forma independiente a las indicaciones médicas, pues debe gestionar en la práctica entre el alivio psicopatológico y los efectos secundarios que amenazan con desintegrar su vida psíquica, física o social. Al actuar de este modo, se evidencia la complejidad de integrar en las diferentes prácticas de estos sujetos los neurolépticos y sus dosis. Se pone de manifiesto lo que Mol^42^ denomina como *política ontológica*, por cuanto obliga a definir qué versión de la realidad debe prevalecer y cuáles son las implicancias de esa elección.

### Neurolépticos y estigma en la inclusión laboral

Las tensiones sociales con el uso de neurolépticos muestran que también tienen un rol relevante en la construcción del estigma. Como hemos visto, el uso de fármacos de forma cotidiana puede traducirse en diversos problemas corporales, psíquicos y sociales. Con relación a este último, las personas participantes refieren que los neurolépticos dificultan la invisibilización del diagnóstico y, por tanto, las puede exponer a desacreditación, tal como se ha descrito en investigaciones en Chile y fuera del país^1^^,^^63^^,^^64^. 

En primer lugar, el uso de fármacos es vivido como una marca de la enfermedad, en tanto su uso les indica que son personas enfermas de esquizofrenia y que su gravedad se correlaciona con la cantidad de fármaco que consumen. Francisco refiere que le gustaría tomar menos medicamentos porque se sentiría más sano tomando menos. Tiene la expectativa que bajando la dosis pueda estar más alerta y con más motivación. Cuenta que una compañera de trabajo toma solo una pastilla, mientras que él toma tres y media de clozapina, además de Ranitidina para el estómago (para contrarrestar el efecto secundario del otro fármaco): “*Me gustaría sentirme mejor, antes yo tomaba seis de clozapina. El problema que tengo es mucha saliva*” (Francisco, entrevista del 17-11-2022).

En la misma línea, al salir de un hogar protegido psiquiátrico en el que visitamos a su hermano, Pedro me dice que quiere dejar los fármacos psiquiátricos porque no se siente enfermo, que está bien y ha logrado hacer una vida normal, que no cree tener esquizofrenia, entonces no ve la necesidad de seguir tomándolos. Me cuenta que ha ido bajando la dosis de a poco y que no ha tenido ningún problema (Observación del 5-1-2023, en hogar protegido).

Por otro lado, el neuroléptico opera como una marca para otras personas, pues devela la presencia de una enfermedad e impide que las personas puedan esconder el diagnóstico y evitar la desacreditación. Marisol se encontraba buscando trabajo por su cuenta, había asistido a una entrevista grupal en la que fue seleccionada. Entonces la llamaron para que firmara contrato. En la entrevista, además de preguntarle por las medidas del uniforme, talla de zapatos, le consultan si toma algún medicamento, a lo que ella responde que toma quetiapina, le preguntan por el diagnóstico y ella dice que es esquizofrenia:

“*Me estaban preguntando cosas sobre los trabajos, estábamos casi firmando el contrato para entrar a trabajar, me estaban pidiendo la talla de la polera, del pantalón, todo. Y va y me dice el caballero ‘No señorita, no la vamos a atender, porque usted tiene que atenderse por SENADIS, que es para las personas discapacitadas’. Y eso a mí me dio mucha pena y mucha lata porque eso es discriminación. Hasta yo con el caballero me puse a discutir, porque me estaba diciendo que las pastillas de quetiapina de 100mg es un somnífero. No es un somnífero, es un antipsicótico. No es un somnífero, es un antipsicótico. Que las personas toman las pastillas que empiezan con ina, ina como clozapina, quetapina, muchas ina, son antisicóticos no somníferos*”. (Marisol, entrevista del 2-3-2022)

Muchos de estos fármacos se deben administrar, por lo general, en diferentes momentos del día, a menudo en tres horarios (mañana, medio día y anochecer). Entonces, el momento en que estas personas están más expuestas es al medio día porque se encuentran dentro sus jornadas laborales o interactuando con otras personas, por lo que deben desarrollar estrategias para evitar develar su uso. En una observación con Marisol fuimos a un café:

“*Marisol le pide un vaso de agua a la persona que nos estaba atendiendo. Le dice que es porque le duele la cabeza, pero me hace un gesto, haciéndome notar que no es esa la razón. Luego me comenta que es uno de los neurolépticos que se tiene que tomar, la quetiapina. Me cuenta que en el trabajo también dice que le duele la cabeza. Sus compañeros de trabajo le han dicho: ‘pero qué rara tu pastilla y qué raro que te duela la cabeza todos los días’. Marisol me afirma que nunca va a contar de su diagnóstico en el trabajo, así como lo ha hecho uno de sus amigos también diagnosticado de esquizofrenia. Dice que no quiere que la traten distinta ni la discriminen. Así que no tiene razón para decir qué pastilla toma ni contar de la esquizofrenia*”. (Observación del 8-6-23)

Situaciones similares refieren a otras personas participantes, quienes esconden el uso de medicamentos para evitar preguntas que las puedan llevar a develar su diagnóstico. Cristián expresa muy claramente el sentir de muchos de los participantes:

“*La esquizofrenia no conviene decírselo a una persona cualquiera. Si estoy trabajando en una parte, el jefe, el empleador lo sabe y si es profesional de área de salud me va a comprender más todavía, pero si es una persona que no sabe nada, si es una persona que no tiene conocimiento, no me comprenda, entonces se va a pasar muchos rollos y me va a poner problemas… Que voy a cometer una locura… Golpear a una persona, hacer destrozos, eso piensan algunas personas ignorantes, eso o se asustan también, hay algunas que se asustan. Pero yo nunca he hecho esas cosas, me encuentro en mi sano juicio*”. (Cristián, entrevista del 25-5-2023)

Además de operar en la cotidianeidad como marca del estigma, en el mundo del trabajo los neurolépticos cumplen un rol complejo, como objeto-frontera^65^, pues habitan la intersección de varias comunidades de práctica y satisfacen las necesidades de información de cada una de ellas. Esto ocurre porque el fármaco no solo pertenece al mundo médico, sino que es considerado como requisito para trabajar, pues funciona como indicador de que la persona se encuentra en tratamiento. 

La asociación entre esquizofrenia y la exigencia de llevar tratamiento farmacológico es generalizada, no solo los equipos médicos incentivan el uso de psicofármacos, sino también las empresas que contratan por inclusión y las fundaciones que buscan la colocación laboral como forma de asegurar que pueden trabajar.

Esto se observa en prácticas que se han establecido para la contratación por inclusión, como solicitar certificados del médico tratante de que están en condiciones para trabajar o revisar el uso de fármacos:

“*Mira, el informe de autorización médica es cuando yo tengo muchas sospechas de que la persona todavía no está preparada para un contexto laboral. Si la persona nos indica que tiene control cada seis meses o cada un año, eso ya me da un índice que ya no necesita tanto apoyo médico. Ahora hay que apoyarlo también dentro de la sociedad, observo que conductualmente, la evaluación o alguna actividad, es una persona normal, como cualquier otra, no necesariamente vamos a solicitar, o yo personalmente voy a solicitar, una autorización médica. Solo en el caso de que la persona me dijera ‘tengo control todas las semanas’, ‘tengo que ir a buscar la receta para los medicamentos’, le digo, ‘ok, ¿sabe tu médico que tú estás interesado en trabajar?*’” (Fundación CD, entrevista del 6-7-2022)

A pesar de que en Chile se ha avanzado en políticas públicas orientadas a la inclusión laboral de personas en situación de discapacidad desde el enfoque de derechos y la mirada social de la discapacidad^66^^,^^67^, la centralidad del criterio médico para distinguir si la persona puede trabajar ha hecho que muchos procesos de intermediación laboral se enfoquen en su condición de salud, más que en sus capacidades y, por tanto, se transforma en central la presencia del neuroléptico:

“*El estigma ha sido la principal dificultad que, en este caso, las personas con esquizofrenia han vivido al momento de enfrentarse a un proceso de intermediación laboral. Y es porque el proceso se centra mucho en su condición de salud y en su toma de medicamentos y estabilización farmacológica y poco se sienten los ajustes y condiciones que requieren para poder hacer un proceso exitoso de capacitación, inducción, desempeño de tareas, etc*.” (Secretaría Nacional de Discapacidad, entrevista del 10-8-2022)

Desde la Fundación P. refieren que dentro del mundo de la inclusión se han incorpo­rado muchos profesionales provenientes del mundo de la salud, que centran su trabajo principalmente en hacer adecuaciones desde el ámbito médico. Por tal razón, se preocupan de que las personas asistan a controles médi­cos y tomen sus medicamentos:

“*Existen como cinco leyes distintas de por qué tu empleador no tendría por qué preguntarte ningún dato médico, pero, sin embargo, con las personas con discapacidad, sí se pregunta mucha información médica ¿Por qué tu empleador tendría que saber cuál es tu médico de cabecera? ¿Cuáles son las medicaciones que te estás tomando y en qué dosis?* [...] *nuevamente se va generando esta medicalización del espacio de trabajo*”. (Entrevista del 13-10-22)

De este modo, cualquier iniciativa para dejar o disminuir el uso de fármacos es restringida por un control social desde diferentes acto­res, supeditando el acceso al trabajo a su uso cotidiano.

Los neurolépticos como objetos frontera permiten que diferentes actores, en distintas prácticas, se articulen con las personas diagnosticadas de esquizofrenia a través de ellos, de modo que se transforman en un importante actante en sus prácticas cotidianas. Estos objetos transforman en marca visible la invisibilidad de la esquizofrenia. Por un lado, tomar neurolépticos se constituye en una práctica que les recuerda y les hace actuar la enfermedad, aunque sientan que no la tienen, pero, también, por otro lado, los mismos fármacos dificultan que puedan esconder el diagnóstico y evitar la desacreditación. Resulta significativo que esta marca se transforme también en requisito para la inclusión laboral, como objeto-frontera que permite la coordinación entre el mundo del trabajo y el mundo médico, consolidando su uso, su carga estigmatizante y la existencia de la tensión psíquica, corporal y social que mediatiza.

## Discusión

Los hallazgos de esta investigación muestran que el uso de neurolépticos por parte de personas trabajadoras diagnosticadas de esquizofrenia se vive como una experiencia compleja con múltiples dimensiones. Los neurolépticos actúan como actantes en las prácticas cotidianas debido a su ensamblaje con el diagnóstico de esquizofrenia, mediando en los ámbitos corporales, psíquicos y sociales de estas personas. Esta complejidad contrasta con la simplificación de la perspectiva biomédica predominante en los tratamientos en Chile, que considera la esquizofrenia como una enfermedad crónica de origen biológico, cuyo tratamiento principal debe ser farmacológico^25^^,^^68^.

Los resultados muestran que se sostiene una práctica en la que el diagnóstico de esquizofrenia implica el tratamiento con neurolépticos de por vida, sin posibilidad de alta ni curación. Esta práctica, anclada en dispositivos de salud que promueven la adherencia constante a la medicación, ha sido documentada como parte de un ejercicio de poder que sitúa a las personas como pacientes en una relación de dependencia con el sistema psiquiátrico^25^. Investigaciones etnográficas previas muestran cómo este encuadre produce una internalización del estatus de “enfermo mental crónico”, afectando la autopercepción, la agencia y la posibilidad de recuperación^32^.

A pesar de que los fármacos son valorados por aliviar síntomas en las fases agudas^21^, su uso se vincula a numerosos efectos secundarios que afectan significativamente la funcionalidad diaria, como somnolencia, problemas gastrointestinales, efectos motores, aplanamiento afectivo y problemas metabólicos^20^^,^^22^^,^^69^. Estos efectos fueron experimentados por las personas participantes de esta investigación, mostrando cómo dificultan mantener un ritmo laboral, estudiar o sostener vínculos sociales. La literatura coincide en señalar que la carga de efectos adversos compromete la adherencia a tratamientos a largo plazo^23^^,^^37^ y algunos estudios reportan que un porcentaje importante de pacientes abandonan los tratamientos por esta razón^22^.

Esta tensión entre la necesidad percibida de medicación y los efectos secundarios ha sido conceptualizada como un “laberinto sin salida”^21^, donde las personas desarrollan estrategias para regular los efectos negativos: ajustes de dosis, horarios o incluso interrupciones del tratamiento, a menudo sin aprobación médica. Tales estrategias también se identificaron en esta investigación, revelando la agencia de las personas frente al mandato de adherencia y la búsqueda de equilibrio entre evitar una crisis y llevar una vida vivible.

Los resultados muestran que los fármacos pueden operar como actantes en el estigma hacia estas personas, pues se transforman en una marca visible y produce efectos visibles que las hace, como señala Goffman^47^, desacreditables. 

En términos sociales y laborales, los efectos visibles del tratamiento farmacológico (temblores, salivación, somnolencia, entre otros) se convierten en nuevos atributos llamativos para otras personas que los exponen a la desacreditación, junto con ser una marca en sí misma que dificulta el ocultamiento del diagnóstico, pues el fármaco es exigido para trabajar y se controla en los procesos de inclusión laboral. Este hallazgo coincide con múltiples estudios que señalan cómo los psicofármacos no solo impactan sobre la salud física y emocional, sino también generan autoestigma y rechazo social^23^^,^^38^. Tal como se ha señalado, el estigma asociado al uso de antipsicóticos compromete seriamente el acceso al empleo y el mantenimiento de los vínculos sociales^15^^,^^26^.

En línea con las críticas que emergen desde los movimientos de sobrevivientes de la psiquiatría y las propuestas del modelo de recuperación, estos hallazgos ponen en cuestión la hegemonía de los tratamientos centrados exclusivamente en la farmacoterapia^70^^,^^71^, pues no consideran las múltiples implicancias de su uso cotidiano.

Este asunto resulta aún más significativo, pues en el ámbito del trabajo y la inclusión laboral, Chile ha avanzado normativamente en el enfoque de derechos para personas con discapacidad, como lo establece la Ley 21015, sin embargo, en la práctica, los dispositivos de inclusión laboral para esta población operan bajo la lógica de continuidad de tratamientos médicos farmacológicos, subordinando los apoyos laborales a criterios médicos de compensación. Esto reproduce la medicalización del trabajo y restringe la autonomía de los sujetos.

Finalmente, los resultados tensionan los supuestos de la literatura dominante que asocia éxito terapéutico con adherencia a neurolépticos y participación en el mercado laboral^8^^,^^11^. Aunque algunos estudios muestran que los psicofármacos podrían favorecer la inclusión laboral, otras investigaciones señalan que no existe correlación entre el alivio sintomático y el bienestar subjetivo^20^ y que el estigma y los factores sociales tienen un peso decisivo en las posibilidades de trabajar^14^. De hecho, investigaciones recientes^28^^,^^34^ han evidenciado que las personas que dejan los fármacos, después de la fase aguda, pueden tener mejores resultados funcionales y menos hospitalizaciones.

Esta investigación enfatiza que los neurolépticos ocupan un lugar clave, de actantes, en las experiencias de estas personas, un lugar complejo y en tensión que, si bien son vividos como una ayuda para la angustia, afectan las experiencias corporales, psíquicas y sociales y operan como marca que devela el diagnóstico y expone al estigma, produciendo importantes dificultades para la inclusión laboral.

Estos hallazgos refuerzan la necesidad de avanzar hacia estrategias de atención que no solo consideren la estabilización farmacológica, sino también las experiencias subjetivas y los contextos sociales en los que las personas trabajadoras diagnosticadas de esquizofrenia intentan construir sus vidas.

## Conclusiones

Las conclusiones de esta investigación permiten responder a la pregunta sobre el lugar de los neurolépticos en las experiencias de personas trabajadoras diagnosticadas de esquizofrenia en relación con su inclusión laboral. Los resultados muestran que los neurolépticos son actantes relevantes en las experiencias de estas personas, son valorados por su efecto en la reducción de síntomas, pero su uso prolongado conlleva importantes tensiones: efectos secundarios físicos, emocionales y sociales que dificultan la vida cotidiana, exponen al estigma y comprometen las posibilidades de inserción laboral. Esta investigación aporta al campo al visibilizar las experiencias subjetivas con el uso de psicofármacos, usualmente ausentes en los estudios centrados en la eficacia clínica. Además, problematiza la hegemonía del enfoque biomédico en los programas de inclusión laboral, donde el tratamiento farmacológico opera como requisito excluyente para trabajar. Se requiere avanzar en modelos de atención que reconozcan la voz de las personas usuarias y permitan mayor autonomía en la gestión de sus tratamientos. 

Respecto a las limitaciones de este estudio, y si­guiendo los criterios de rigor de Guba y Lincoln^72^, se reconoce que la transferibilidad de los hallazgos -es decir, que puedan ser aplicados en otros contextos- está supeditada a las condiciones socioeconómicas y culturales espe­cíficas de las personas participantes.

Investigaciones futuras podrían explorar experiencias de reducción o suspensión de fármacos en contextos laborales, así como experiencias de inclusión desde enfoques no farmacológicos, contribuyendo a repensar políticas de salud mental y empleo desde un enfoque de derechos. Asimismo, se sugiere explorar los factores socioeconómicos en la experiencia de uso de neurolépticos.
