# Supplementary material for: Inclusión laboral y medicalización: neurolépticos como actantes en las experiencias de personas trabajadoras diagnosticadas de esquizofrenia en Chile
Source: Salud Colect. 2026 Mar 10;22:e5903. [Article in Spanish] doi: 10.18294/sc.2026.5903 (PMC13451015; doi:10.18294/sc.2026.5903)
Supplement: Translation

# Translation: Labor inclusion and medicalization: neuroleptics as actants in the experiences of workers diagnosed with schizophrenia in Chile

**Authors:** Ernesto Bouey Vargas, María José Reyes Andreani

**Affiliations:** 1 PhD in Social Sciences. Professor, Faculty of Psychology, Universidad Diego Portales, Santiago, Chile. ernesto.bouey@mail.udp.cl; 2 PhD in Social Psychology. Academic, Department of Psychology, Universidad de Chile, Santiago, Chile. mjrandreani@u.uchile.cl

**Keywords:** Schizophrenia, Antipsychotic Agents, Working Conditions, Social Stigma, Chile

## Abstract

People diagnosed with schizophrenia exhibit low rates of labor market participation, a phenomenon that has been predominantly addressed from biomedical approaches that associate work inclusion with pharmacological stabilization. However, there is limited research examining how the everyday use of neuroleptics shapes the work experiences of those employed with this diagnosis. The aim of this study was to understand the role of neuroleptics - conceived as actants - in the experiences of workers diagnosed with schizophrenia in Chile and their implications for labor inclusion. A qualitative ethnographic study was conducted between 2022 and 2024 in the Metropolitan Region of Chile, including participant observation across diverse everyday and workplace settings, as well as 86 interviews (53 with workers diagnosed with schizophrenia and 33 with individuals from their close environment). Drawing on actor-network theory and the notion of enactment, the analysis identified that the diagnosis and the neuroleptic become assembled as a lifelong practice, configuring an experience marked by tensions. Although medications are valued for alleviating distress, their assemblage with diagnosis and their side effects - sedation, motor disturbances, salivation, weight gain, and affective flattening - mediate the body, the psyche, and social life, hindering work performance and exposing individuals to stigma and discrimination.

## Introduction

People diagnosed with schizophrenia constitute one of the most socially excluded groups worldwide. Numerous studies have shown that their rates of labor market participation are extremely low, with estimates ranging between 4% and 10%, although they may reach up to 50% within specific employment programs in countries of the Global North.^1^^,^^2^ In Chile, although there are no data that allow for establishing a precise employment rate for this population, a review of public databases suggests employment rates ranging between 19% and 3%,^3^^,^^4^ depending on the measurement methodology used. This exclusion becomes even more significant when considering that employment has been identified as a fundamental element for recovery, improved well-being, self-esteem, and social inclusion among people who have received this diagnosis.^5^^,^^6^^,^^7^

Research on schizophrenia and employment has historically been dominated by biomedical and psychopathological approaches. From these perspectives, access to work has been conceptualized as the result of clinical stabilization, primarily achieved through prolonged pharmacological treatments.^8^^,^^9^^,^^10^ In this line, studies have sought to identify those medications that most effectively facilitate entry into or retention in the labor market.^11^ This perspective has been reinforced by the development of programs such as Supported Employment and Individual Placement and Support, in which adherence to pharmacological treatment plays a central role in labor inclusion.^12^^,^^13^

However, several studies have pointed out that this perspective is limited in explaining processes of labor inclusion. In particular, for more than two decades it has been argued that low employability cannot be explained solely by the clinical condition and its treatment; rather, it must be understood through the intersection of social, economic, contextual, family, and educational factors.^2^^,^^14^ In this regard, stigma has been identified as one of the main barriers, affecting both the possibility of obtaining and maintaining employment.^15^^,^^16^^,^^17^ These studies emphasize that, more than the clinical condition and its psychopathological manifestations, labor inclusion is mediated by social contexts.

One aspect that has received less attention is the role played by pharmacological treatments in these processes. Historically, their widespread use for the treatment of schizophrenia dates back to the 1950s and 1960s, in the context of the “pharmacological revolution” marked by the emergence of chlorpromazine, whose main effects were sedation and a reduced interest in both the environment and oneself.^18^ The term neuroleptic was coined because therapeutic efficacy was associated with observable neurological effects, such as muscle spasms, restlessness, and tremors, among others. Later, so-called second-generation treatments emerged with compounds such as clozapine. Over time, the concept of neuroleptic was replaced by that of antipsychotic, reflecting a shift toward a disease-centered model in which medications were assumed to act on the underlying biological processes of mental illnesses such as schizophrenia, correcting chemical imbalances in the brain. This new conceptualization rendered the relevance of neurological effects largely invisible in both clinical practice and research.^19^ For this reason, in this study we will preferentially use the term neuroleptic, since a central focus of analysis concerns experiences related to side effects.

In this regard, studies have shown that second-generation antipsychotics present side effects that negatively affect people’s social and functional lives.^20^^,^^21^ These include sedation, cognitive dysfunction, weight gain, sexual dysfunction, emotional blunting, reduced concentration capacity, tremors, and motor disturbances.^22^^,^^23^^,^^24^^,^^25^^,^^26^^,^^27^^,^^28^ They have also been associated with metabolic problems that may lead to different cognitive deficits,^29^ as well as weight gain, hyperglycemia, and dyslipidemia,^30^ increasing the likelihood of developing diabetes.^31^

Qualitative and ethnographic studies have further shown that neuroleptics not only affect physical and functional dimensions but also shape the construction of personal identity, self-perception, and ways of relating to the surrounding environment. In a pioneering study, Sue E. Estroff^32^ demonstrated how the effects of psychiatric medications influenced how individuals came to conceive of themselves as “mentally ill,” reinforcing processes of stigmatization and the internalization of the diagnosis. Subsequent research has reinforced this perspective by showing that prolonged pharmacological treatment can generate experiences of devitalization, affective flattening, and a diminished sense of personal agency.^25^^,^^26^^,^^33^

More recently, the assumption that pharmacological treatment should necessarily be indefinite has begun to be questioned. Longitudinal studies indicate that, after the first years following diagnosis, many individuals may reduce or discontinue neuroleptic medication without experiencing worse prognoses, and in some cases achieving better levels of functional recovery.^28^^,^^34^ Nevertheless, those who attempt to discontinue or reduce medication face multiple barriers, ranging from fears of relapse conveyed by health professionals to the absence of institutional support to navigate this process.^21^^,^^35^

These dynamics operate both within the medical field and in labour intermediation processes. For instance, individuals may be required to provide certificates of “clinical stability” or proof of psychotropic medication use as a condition for accessing employment. Such requirements reproduce a logic of medical surveillance over the working lives of people diagnosed with schizophrenia.^27^^,^^36^

These antecedents reveal a largely unexplored tension between the use of neuroleptics and processes of access to employment. Although a critical body of literature has examined the adverse effects of psychotropic medications and the subjective experiences associated with their use,^37^^,^^38^ in-depth studies addressing the experiences of workers diagnosed with schizophrenia in relation to the use of neuroleptics remain largely absent. In particular, little attention has been paid to how these experiences intersect with the opportunities and barriers that shape their labour inclusion.

Conceptually, this study analyses the role of neuroleptics as agents in the social lives of these individuals. Following Bruno Latour,^39^ non-human elements may also possess agency (*actants*), insofar as, once inserted into a network, they assemble with other elements and modify the course of action. From this perspective, the place of neuroleptics will be examined through the experiences of people diagnosed with schizophrenia.

Annemarie Mol,^40^^,^^41^ through the concept of enactment, argues that different elements - such as objects, bodies, and disease - can be assembled with one another, being produced and acquiring reality within specific practices. As a result, they become multiple depending on the activities in which they participate. In this sense, neuroleptics may become different things within different networks of practice.^42^ For the author,^41^ bodies are not closed wholes but have semi-permeable boundaries, incorporating external objects in order to function. For example, food (such as an apple) moves from being external to becoming part of internal stability, much like medications. Mol refers to a metabolic body whose paradigmatic activity is metabolizing (eating, breathing, excreting), emphasizing that its boundaries are fluid and that continuous exchange with the environment is essential - particularly, in this case, with pharmaceuticals. This conceptualization allows us to understand bodies as configurations of tensions that must be managed.

As several studies have shown,^43^^,^^44^^,^^45^^,^^46^ workers diagnosed with schizophrenia must deal with social stigma in their everyday lives, and neuroleptics play a role within these practices. Following Erving Goffman,^47^ stigma is defined as a relational process in which an individual is identified through an arbitrary attribute that differentiates them from others, turning them into a discredited or potentially discreditable person and exposing them to discrimination. Stigma has consequences for the identity of these individuals, who may internalize it, resist it, conceal the mark, or seek support from others. In relation to stigma associated with schizophrenia (which Goffman describes as a *defect of character*), because it is not immediately perceptible, individuals may attempt to conceal and manage this mark in order to avoid discreditation. In this sense, stigma is not inherent to the person but rather constitutes a social mark imposed upon them. Consequently, the diagnosis of schizophrenia itself becomes part of the stigmatizing process: a mark that can lead to discreditation.^48^^,^^49^

Building on these empirical and conceptual antecedents, this study aims to examine in depth the experiences associated with the use of neuroleptics - understood as *actants* - among workers diagnosed with schizophrenia and the implications of these experiences for their labour inclusion.

## Methodology

This research was conducted using a qualitative ethnographic approach aimed at understanding the lived experiences of workers diagnosed with schizophrenia, paying particular attention to their meanings and everyday practices.^50^^,^^51^^,^^52^ An inductive logic guided the research process,^53^ situating participants’ narratives within their broader interpretive and contextual frameworks.^54^

Participants were selected based on territorial criteria (Metropolitan Region of Chile), clinical criteria (a diagnosis of schizophrenia), and employment criteria (having held formal employment during the previous two years). The selection process combined theoretical sampling^55^^,^^56^ with convenience sampling and also drew on snowball sampling techniques.^54^ Participants were contacted through employment placement organizations, mental health professionals, and acquaintances of the principal investigator. These intermediaries explained the characteristics of the study and provided the researcher’s contact information so that individuals could voluntarily participate in the research. The final sample consisted of 16 individuals diagnosed with schizophrenia with recent formal work experience (Table 1), as well as 30 people from their close social environment, including family members, co-workers, direct supervisors, mental health professionals, and staff working in labour inclusion programs.

**Table 1:** Characteristics of workers diagnosed with schizophrenia who participated in the study. Chile, 2022-2024.

Pseudonym	Age	Gender	Educational level	Main neuroleptic	Disability pension
Francisco	45	Male	Incomplete higher education (technical)	Clozapine	Yes
Pablo	52	Male	Incomplete higher education (university)	Clozapine	Yes
Cristián	33	Male	Secondary education	Clozapine	Yes
Enrique	34	Male	Higher education (technical)	Clozapine	Yes
Pedro	57	Male	Primary education	Risperidone	Yes
Martín	41	Male	Higher education (university)	Aripiprazole	No
Irene	45	Female	Primary education	Clozapine	Yes
Osvaldo	39	Male	Incomplete higher education (university)	Olanzapine	Yes
Marisol	27	Female	Incomplete primary education	Quetiapine	Yes
Rocío	51	Female	Higher education (university)	Clozapine	No
Diego	43	Male	Primary education	Quetiapine	Yes
Pamela	33	Female	Incomplete secondary education	Clozapine	Yes
Lucas	34	Male	Secondary education	Clozapine	Yes
Nicolás	40	Male	Incomplete secondary education	Clozapine	Yes
Rodolfo	53	Male	Incomplete secondary education	Clozapine	Yes
Sonia	59	Female	Secondary education	Aripiprazole	Yes

Source: Own elaboration. Note: The Chilean education system is structured as follows: Early childhood education, primary education (1st to 8th grade), secondary education (9th to 12th grade), and higher education (universities, professional institutes, and technical training centers).

The techniques employed were participant observation and ethnographic interviews. Observation took place across multiple settings defined by the everyday activities of the participants, incorporating mobility and the multiplicity of spaces involved in their daily lives.^57^^,^^58^ In this sense, the researcher accompanied participants in their daily activities to the extent that they wished to be accompanied. The observed settings included medical appointments, various forms of travel and commuting, supported housing facilities, job-search sites such as the Municipal Office for Labour Information *(Oficina Municipal de Información Laboral, OMIL)* or foundations, public services, workplaces, among others.

Between 2022 and 2024, 32 detailed field reports were produced, integrating thick descriptions, participants’ interpretations, theoretical reflections, and methodological considerations.^59^^,^^60^ The ethnographic interviews were non-directive in nature and sought to foster reflexivity and the subjective expression of participants’ experiences.^51^^,^^61^ A total of 86 interviews were recorded and transcribed: 53 with workers diagnosed with schizophrenia and 33 with individuals from their social environment. These interviews were conducted during participant observation and therefore took place in the various settings described above. Most were individual interviews, although some collective interviews were also carried out. Participation was voluntary and confidentiality was assured.

Data analysis occurred simultaneously with data production, following a recursive logic consistent with ethnographic research and constructivist grounded theory.^53^^,^^62^ The software NVivo was used to organize the material. Coding began with an open coding process in which the research team inductively developed codes, relating them to one another and constructing emerging categories. During this process, hypotheses were generated and subsequently tested or rejected through ongoing data analysis and fieldwork with participants (theoretical sampling). As categories and their relationships were progressively consolidated through axial coding, new hypotheses continued to be developed and discussed with participants until theoretical saturation was reached. Finally, selective coding was carried out to structure the findings concerning experiences with neuroleptics in relation to access to employment.

The study received approval from the Research Ethics Committee of the Faculty of Social Sciences at the University of Chile (Report No. 25-27/2022), the institution hosting the research. All participants were informed about the characteristics of the study and signed written informed consent forms guaranteeing confidentiality, voluntary participation, and the protection of participants’ identities through the use of pseudonyms and the anonymization of other individuals and locations.

## Results

### The lifelong assemblage of schizophrenia diagnosis and neuroleptics

One aspect that strongly shapes the lives of people diagnosed with schizophrenia is the message they receive that the diagnosis is lifelong. This means that they will never be medically discharged nor told that they have been cured or recovered, implying that they must continue taking medication for the rest of their lives. Nicolás explains:

“*I’m very clear that this has no cure at the moment. There’s no cure for schizophrenia, that’s pretty clear. And I have to keep taking the medications, be responsible, because I know very well that if I stop taking them there’ll come a point when my body’s going to need them, my brain too, and I’ll decompensate, I’ll end up hospitalized.*” (Nicolás, interview, 2023-07-17)

Enrique expressed a similar view:

Researcher: “*You were telling me that a schizophrenia diagnosis lasts a lifetime, that they can’t discharge you.*”

Enrique: “*Yeah. It’s because you have to keep taking the medications all the time…*” (Enrique, interview, 2023-01-24*)*

All the workers diagnosed with schizophrenia who participated in this study were undergoing pharmacological treatment. Likewise, the mental health professionals who took part in the research maintained that pharmacological treatment is essential for managing schizophrenia, arguing that if patients discontinue it they tend to experience psychotic relapses.

The diagnosis establishes a new everyday practice consisting of taking medication three times a day: in the morning, at midday, and in the evening. In some cases, so-called depot neuroleptics are administered, which are injected once a month in patients who have difficulty adhering to treatment. However, all participants in this study maintained the routine of taking oral medication three times a day. In this way, the diagnosis and neuroleptics become a lifelong assemblage in the lives of people diagnosed with schizophrenia - one that is sustained daily through medical prescription.

### Tensions surrounding the use of psychotropic medication

Although the participants in this study continued attending psychiatric appointments and taking their medications, many expressed a marked ambivalence regarding their use. On the one hand, some participants reported that their current treatments helped them feel better, particularly by reducing anxiety and distress. In general, they expressed gratitude toward the medications, identifying them as responsible for their improvement, and some hoped they would continue helping them as they had so far. For example, Pablo explained:

“*With this medication I feel pretty relaxed. It lets me stay consistent with what I do. In a job I can keep steady, or if I’m studying it helps me stick with it and get up early to work - it doesn’t feel that heavy.*” (Pablo, interview, 2023-05-25)

On the other hand, some participants expressed a desire to stop taking neuroleptics, stating that they felt well and did not believe they needed them. This desire was related both to avoiding the side effects associated with the medication and to the idea that discontinuing it would reaffirm that they were healthy and no longer required treatment. Osvaldo commented:

“*I’d love to take fewer pills and feel good, healthy, yeah. But if it can’t be done, then whatever.* […] *Yeah, yeah, it’s not something you can really do much about when you’re taking the meds-it’s just side effects. You’ve got to go with the lesser evil, something like that*.” (Osvaldo, interview, 2023-07-13)

Field observations reflected similar tensions. For instance:

“*Pedro, on the other hand, repeatedly insisted that he wanted to stop taking his medication because he felt well, but the doctors kept telling him that he should not discontinue the treatment.*” (Field observation, 2023-01-05)

In this way, the assemblage between the diagnosis and neuroleptics is experienced by participants as a source of tension. While the medication helps reduce anxiety and distress, it also produces suffering due to its side effects and to the meaning associated with being identified as a person with a mental illness.

#### Neuroleptics as mediators between the body, psyche, and social life

The diagnosis/neuroleptic assemblage operates as a mediator^39^ in the experiences of people diagnosed with schizophrenia, insofar as it significantly transforms them.

It is common to hear accounts of people gaining weight due to psychiatric medications. For example, Osvaldo (interview, 2023-07-13) mentioned that he is now at a healthy weight, but that he once reached 93 kilograms as a result of the medication. In general, problems derived from antipsychotics directly affect participants’ everyday lives. Cristián, for instance, explained that the medication causes him stomach problems:

“*With clozapine the issue was my stomach, because my stomach’s really sensitive. I can eat just about anything and it messes me up.* […]. *Before I started taking the pill it wasn’t like that. I could eat cheese or drink milk. Now milk, dairy, all that stuff makes me feel bad.*” (Cristián, interview, 2023-05-04)

Other bodily effects were also reported. During a visit to a supported housing facility, one of the caregivers commented:

“*Clozapine, for example, can give you tachycardia. I don’t know if you noticed that* [name of a resident] *has kind of a bluish-purple color to his skin when he’s around here. He’s got a heart issue. They’ve lowered his clozapine because it gives him tachycardia. It also causes constipation.* [Name of another resident], *for example, struggles all week to go to the bathroom because of the medication. There are other medications that keep them like this, shaking.* […] *And that clearly becomes a barrier to going out.”* (Caregiver, interview, 2023-08-24)

Pablo also recounted that he experienced dystonia (involuntary muscle contractions) as a result of a medication he received when he was 19 years old:

“*What causes dystonia is Haldol*^®^ *.* […]. *I was about nineteen or twenty and the doctor gave me Haldol*^*®*^ *and it gave me dystonia, but to counteract it he prescribed Tonaril*^*®*^ .” (Pablo, interview, 2023-05-25)

Marisol mentioned on several occasions how distressing it was for her whenever she experienced dystonia. In her case, the manifestation involved her eyes rolling upward and the muscles around them becoming tense. She described that when this happens she cannot do anything else, because she cannot see properly. In addition, the people around her would become frightened and react with rejection. For this reason, for several months she avoided leaving her home in order to prevent such episodes from occurring in public without anyone available to help her.

Motor symptoms are not limited to dystonia. During an observation conducted in a psychiatric supported housing facility on 25-05-2023, extreme rigidity in the bodily movements of many residents was noted:

“*…what strikes me is the stiffness in the bodies of some people with schizophrenia living in the home. A man walks past me without lifting his head, arms hanging at his sides, no swing in them, and a facial expression somewhere between absent and surprised.*” (Field observation, 2023-05-25, supported housing facility)

They walk without moving their hands, some with their heads lowered and with mechanical movements:

“*A man around thirty walks by accompanied by two staff members, a man and a woman, probably nursing assistants or nurses. They’re holding him by one arm. His movements look very rigid, head down, like he can’t bend his knees or move his arms freely. Marisol keeps watching, and when he leaves she tells me it makes her really sad to see that, that when she was hospitalized people would end up like that because of the drugs and the electroshock, and that she was like that too.*” (Field observation, 2022-10-20, with Marisol, psychiatric hospital)

These same types of movements can also be observed inside psychiatric hospitals (Field observations with Enrique on 2023-01-25; with Cristián on 2023-05-04; and with Irene on 2023-07-14).

Participants in this study reported that the effects they have experienced from neuroleptics, particularly when they are acute or more visible, often force them to withdraw and avoid contact with others, including working or searching for jobs. They described medications for schizophrenia as representing a significant barrier to social interaction, frequently leading to isolation. In this regard, Osvaldo explained that the medication causes tremors in his hands, severe enough to affect his social life:

“[The medications] *have side effects. I don’t know if it’s because I take a lot of pills, but my hands shake a lot all the time and it’s really uncomfortable. Like, meeting people who like board games for me is kind of a no - I can’t really move a piece from one place to another without it being obvious that I’m shaking and people seeing that and not getting why it bothers me, you know? Like, the social part.* […] *Right now I’m actually talking with someone who’s a graphic designer and he’s encouraging me to draw, which I used to love - drawing and painting - but because of the tremor I stopped*.” (Osvaldo, interview, 2023-07-13)

Salivation is another highly relevant issue for participants. Several reported either excessive salivation or dry mouth as a result of psychiatric medications. Some explained that they must make an additional effort to prevent saliva from dripping from their mouths, while others need to keep a bottle of water nearby at all times to cope with dryness. Enrique recounted:

“*When I started with the medications, the saliva… risperidone made me stiff. Risperidone made me stiff… the saliva*.” (Enrique, interview, 2025-01-25)

Francisco also mentioned that salivation is what worries him the most, explaining that he drools excessively both during the night and throughout the day (Field observation, 2023-05-05).

For Pablo and Cristián, the problem of salivation is associated with clozapine and occurs mainly at night while they are sleeping. For many others, however, it occurs during the day and does not necessarily appear at the beginning of treatment, sometimes emerging later-about a year after starting medication. Nicolás described it in the following way:

“*Clozapine knocks you out, you start drooling and all that stuff.*” (Nicolás, interview, 2023-07-17)

Participants reported that, because of excessive salivation, they often avoid conversations with others, as they have noticed that it can provoke rejection. However, they cannot control it. Some participants, such as Marisol, Nicolás, or Cristián, have felt compelled to explain to others in workplace contexts - such as job interviews or when speaking with co-workers and supervisors - that this is a side effect of their medication.

Another side effect frequently mentioned is daytime sedation, which significantly affects the search for and attainment of employment. Osvaldo explained that the medication calms him but also leaves him less alert, making him uncertain whether it ultimately helps or hinders him:

“*In fact, when I started getting really nervous at work they gave me clonazepam, which in the end made me calmer but less alert to what was going on. So I didn’t know if it actually helped me for working. I’m not sure if it worked in my favor or against me - I felt better, but I wasn’t functioning better.*” (Osvaldo, interview, 2023-07-13)

Martín commented that he used to feel an exhausting fatigue with his previous medications, but that he can now work longer hours because his current treatment does not exhaust him as much:

“*I mean, yeah, it makes me tired, but I’d have to be, like… with… a lot of sleep deprivation and a lot of work to feel really exhausted. But it’s not that overwhelming fatigue I used to feel before.*” (Martín, interview, 2023-09-22)

Irene reported that neuroleptics make her sleepy, so she always has to make an effort to stay awake. Pamela described a similar experience; for this reason she spoke with her psychiatrist and managed to concentrate most of her dosage at night, so she could take the medication and go to sleep. Even so, she still feels sedated during the day. Lucas explained that he often fell asleep during daily activities and that he lost a job because of this side effect:

“*Honestly, sometimes I would fall asleep because I was taking a lot of pills, a lot of medication, and I’d just fall asleep.*” (Lucas, interview, 2023-01-04)

Nicolás was particularly explicit in describing how this effect of neuroleptics prevented him from carrying out important activities in his life, such as completing training courses aimed at finding employment:

“*When I was taking 750 milligrams of clozapine at night, I was studying at* [a labour inclusion foundation] *the first time, but I couldn’t continue because I was doing really badly, really doped up. I’d get to class and be like this, like this, almost falling asleep, and drooling, like that level. And the teacher told me, ‘Nicolás, I want to talk to you after class.’ And she said, ‘What’s going on with you, Nicolás? You’re not well, you’re drooling, you’re barely paying attention in class.’ It must have been the medications, because at that time I was just somewhere else.*” (Nicolás, interview, 2023-07-17)

In addition to limiting the activities participants wished to pursue, sedation also interfered with their relationships with others. Several participants reported being rejected from jobs because they appeared overly sedated. At the same time, sedation also affected their relationships with friends. Some participants recounted that friends mocked them for falling asleep or being unable to concentrate; others described friendships that gradually faded. At the same time, some friends attempted to support and assist them.

Pamela’s account is particularly significant in this regard. She had moved to live with a friend outside Santiago, felt very happy, and experienced a strong sense of independence. However, her friend began to feel pity for her after seeing how the medications caused sedation:

“*…because she saw me doped up. She’d see me when I took my meds and I’d get a little doped up. And she’d tell me it made her sad to see me like that.*” (Pamela, interview, 2024-01-04)

Another area in which participants reported that the medication exerts agency concerns emotions. Several participants stated that the medication has helped reduce anxiety and distress, but that it also produces emotional effects that lead them to feel less interest in matters that were previously important to them. Martín expressed uncertainty about the origin of this feeling:

“*I’m not very interested in money or material things. Because… I don’t know if… uh, I told you, but… I’m a person who has flattened emotions. Uh, I don’t remember exactly what the symptom is called… But I don’t feel anger, resentment, hatred, joy, sadness - any kind of emotion. They once told me it’s because of the medications. But I’m not going to stop the medications, because I don’t want to relapse into the illness again. They already told me that if it happens a third time, I wouldn’t leave the hospital.*” (Martín, interview, 2023-09-02)

This has also affected his personal life. For example, he explained that problems no longer concern him, that he has even forgotten what his life was like before the diagnosis of schizophrenia, but that he also does not feel any need to remember it. This emotional flattening has even affected his interest in forming romantic relationships:

Martín: “*Before I had* [a partner]… *but after the illness, no… nothing happens anymore, no, nothing happens… if there are no emotions, then even less the other stuff.*”

Researcher: “*What do you mean nothing happens? Don’t you feel attraction?*”

Martín: “*No, nothing, nothing, nothing.*” (Martín, interview, 2023-09-22)

Martín explained that this lack of interest extends across different spheres of his life, including work. He stated that he is not interested in working or earning money and that he works only to avoid being a burden to his father, who takes care of him. He also reflected that this might be one of the reasons why many people diagnosed with schizophrenia do not work or feel interested in doing so.

### Everyday management of neuroleptics

Thus, the diagnosis/neuroleptic assemblage produces tension in the experiences of these individuals. On the one hand, it is understood as necessary because of the diagnosis of schizophrenia; on the other, it becomes an actant that modifies the body, the psyche, and social life, making it difficult - or at times imposible - to carry out everyday life normally and to work. Sonia expresses this tension very clearly:

“*Life isn’t easy either. I mean, with a lot of difficulties, because… waiting for people to accept us, you know… like zombies. Because there’s no other word to describe it. It’s like you’re a zombie. Being… because people have to be doped up* [medicated], *because otherwise they reach the point of just leaving this world. It has to be done. And I didn’t want to leave this world either. But it’s difficult, I mean, always trying to say, no, you can do it, you’re going to make it*.” (Sonia, interview, 2023-08-24)

She values the medications, but must constantly weigh how much the drug helps her and how much it harms her. She explains that the medication allows her to feel calmer in relation to distressing thoughts, but that it also produces strong sedation, preventing her from performing well at work:

“*Yeah, it makes me really sleepy. I mean… besides, I know my body. The doctor doesn’t - ‘take the whole pill,’ something like that. I don’t even remember exactly, but the thing is that I take half of half the pills. So I’m not so sleepy, but also to comply, to be responsible. Because I’m already on this boat… I’m already here. So I have to do what I’m supposed to do and be responsible, more than anything in life. But I… I want to do my part. The only thing I want is to keep going forward. I don’t want to walk around like a zombie either*.” (Sonia, interview, 2023-08-24)

Sonia’s way of managing medication is not unique to her. In different conversations, participants recounted moments when they had to modify medical prescriptions themselves, especially in order to stay more alert, reduce physical side effects, or cope with intense distress.

Understanding that the body is actively constructed, the use of pharmaceuticals cannot be understood as simple passive adherence to a norm. Rather, it constitutes an everyday practice through which individuals actively manage a set of tensions. This management often involves intervening independently of medical prescriptions, as individuals must navigate in practice between the alleviation of psychopathological symptoms and the side effects that threaten to disrupt their psychic, physical, or social lives. In acting this way, the complexity of integrating neuroleptics and their dosages into the various practices of these individuals becomes evident. This dynamic illustrates what Annemarie Mol^42^ describes as *ontological politics*, insofar as it compels actors to define which version of reality should prevail and what the implications of that choice might be.

### Neuroleptics and stigma in workplace inclusion

Social tensions surrounding the use of neuroleptics show that they also play a relevant role in the construction of stigma. As we have seen, the everyday use of medication may translate into diverse bodily, psychological, and social problems. With regard to the latter, participants report that neuroleptics make it difficult to conceal the diagnosis and, therefore, may expose them to discrediting, as has been described in studies conducted in Chile and elsewhere.^1^^,^^63^^,^^64^

First, the use of medication is experienced as a mark of illness, insofar as its use indicates to them that they are people with schizophrenia and that the severity of their condition correlates with the amount of medication they take. Francisco reports that he would like to take fewer medications because he would feel healthier taking less. He expects that by lowering the dose he might feel more alert and more motivated. He explains that a coworker takes only one pill, whereas he takes three and a half pills of clozapine, in addition to ranitidine for his stomach (to counteract the side effect of the other medication): “*I would like to feel better; before I used to take six clozapine tablets. The problem I have is a lot of saliva*” (Francisco, interview, 2022-11-17).

In the same vein, after leaving a psychiatric supported housing facility where we had gone to visit his brother, Pedro tells me that he wants to stop taking psychiatric medication because he does not feel ill, that he is doing well and has managed to build a normal life. He says that he does not believe he has schizophrenia and therefore sees no need to continue taking them. He explains that he has gradually reduced the dose and has not experienced any problems (Observation, 2023-01-05, supported housing facility).

On the other hand, the neuroleptic operates as a marker for others, as it reveals the presence of an illness and prevents people from concealing the diagnosis and avoiding discrediting. Marisol had been looking for work on her own and attended a group interview where she was selected. She was then called to sign the contract. During the interview, in addition to asking her about the size of the uniform and her shoe size, they asked whether she was taking any medication. She replied that she was taking quetiapine; they then asked about her diagnosis, and she said it was schizophrenia:

“*They were asking me things about the job, we were almost signing the contract to start working. They were asking for my T-shirt size, my pants size, everything. And then the man says to me, ‘No miss, we are not going to take you on, because you have to be assisted through SENADIS, which is for people with disabilities.’ And that made me very sad and very upset because that is discrimination. I even started arguing with the man, because he was telling me that the 100 mg quetiapine pills are a sleeping pill. It’s not a sleeping pill, it’s an antipsychotic. It’s not a sleeping pill, it’s an antipsychotic. The pills that start with ‘-ine’, like clozapine, quetiapine, many ‘-ine’ medications, are antipsychotics, not sleeping pills*.” (Marisol, interview, 2022-03-02)

Many of these medications must generally be administered at different times of the day, often three times (morning, midday, and evening). The moment when these individuals are most exposed is at midday because they are within their working hours or interacting with other people, and therefore must develop strategies to avoid revealing their use. During an observation with Marisol we went to a café:

“*Marisol asks the person serving us for a glass of water. She says it is because she has a headache, but she makes a gesture to me, letting me know that this is not the real reason. Later she tells me that it is one of the neuroleptics she has to take, quetiapine. She explains that at work she also says she has a headache. Her coworkers have told her: ‘Your pill is strange, and it’s strange that you have a headache every day.’ Marisol tells me that she will never disclose her diagnosis at work, just as one of her friends who has also been diagnosed with schizophrenia does not. She says she does not want to be treated differently or discriminated against. So she sees no reason to say what pill she takes or to talk about schizophrenia.*” (Observation, 2023-06-08)

Similar situations are reported by other participants, who conceal their use of medication in order to avoid questions that might lead them to disclose their diagnosis. Cristián expresses very clearly the feelings shared by many of the participants:

“*Schizophrenia is not something you should tell just anyone. If I am working somewhere, the boss or employer knows, and if they are a health professional they will understand me even more. But if it is someone who knows nothing, someone without any knowledge, they will not understand me. Then they start imagining things and will create problems for me… That I might do something crazy… Hit someone, destroy things-that’s what some ignorant people think, or they get scared too, some of them get scared. But I have never done those things; I am in my right mind*.” (Cristián, interview, 2023-05-25)

In addition to operating in everyday life as a marker of stigma, neuroleptics play a complex role in the world of work as a boundary object^65^, as they inhabit the intersection of several communities of practice and satisfy the informational needs of each of them. This occurs because the medication does not only belong to the medical sphere but is also considered a requirement for employment, as it functions as an indicator that the person is undergoing treatment.

The association between schizophrenia and the expectation of maintaining pharmacological treatment is widespread. Not only do medical teams encourage the use of psychotropic medication, but companies that hire under workplace inclusion programs and foundations that promote job placement also view treatment adherence as a way of ensuring that individuals are able to work.

This can be observed in practices that have become established in inclusive hiring processes, such as requesting certificates from the treating physician stating that the person is fit to work or reviewing the use of medication:

*“Look, the medical authorization report is requested when I have many suspicions that the person is not yet ready for a work environment. If the person tells us that they have check-ups every six months or once a year, that already gives me an indication that they no longer need as much medical support. Now we also have to support them within society. If, behaviorally, based on the evaluation or some activity, I see that the person is normal, like anyone else, then we will not necessarily request - or at least I personally will not request - a medical authorization. Only in the case that the person tells me ‘I have check-ups every week,’ or ‘I have to go pick up my prescription for the medication,’ then I ask them, ‘Okay, does your doctor know that you are interested in working?*’” (Fundación CD, interview, 2022-07-06)

Although Chile has made progress in public policies aimed at the labour inclusion of persons with disabilities from a rights-based approach and a social model of disability,^66^^,^^67^ the centrality of the medical criterion in determining whether a person is able to work has led many labour intermediation processes to focus on their health condition rather than on their abilities. As a result, the presence of neuroleptics becomes central.

“*Stigma has been the main difficulty that, in this case, people with schizophrenia have experienced when facing a labour intermediation process. This is because the process focuses heavily on their health condition, their medication use, and pharmacological stabilization, while little attention is given to the adjustments and conditions they require in order to successfully go through training, induction, job performance, and related processes*.” (National Disability Secretariat, interview, 2022-08-10)

Representatives from Fundación P. note that within the field of workplace inclusion many professionals from the health sector have been incorporated, and they tend to focus their work primarily on making adjustments from a medical perspective. For this reason, they are concerned with ensuring that individuals attend medical check-ups and take their medication:

“*There are about five different laws explaining why your employer should not ask you for any medical information. However, when it comes to people with disabilities, a great deal of medical information is requested. Why should your employer need to know who your primary doctor is? What medications you are taking and at what dose?* […] *Once again, this leads to the medicalization of the workplace.*” (Interview, 2022-10-13)

In this way, any initiative to stop or reduce medication use becomes constrained by social control exercised by different actors, subordinating access to employment to the daily use of these medications.

As boundary objects, neuroleptics enable different actors, across diverse practices, to engage with people diagnosed with schizophrenia through them, thereby becoming an important actant in their everyday practices. These objects transform the invisibility of schizophrenia into a visible marker. On the one hand, taking neuroleptics becomes a practice that reminds individuals of the illness and compels them to enact it, even when they feel they do not have it. On the other hand, the same medications make it difficult to conceal the diagnosis and avoid discrediting. It is significant that this marker also becomes a requirement for workplace inclusion, functioning as a boundary object that allows coordination between the world of work and the medical sphere, consolidating its use, its stigmatizing burden, and the psychic, bodily, and social tensions that it mediates.

## Discussion

The findings of this study show that the use of neuroleptics by working people diagnosed with schizophrenia is experienced as a complex phenomenon with multiple dimensions. Neuroleptics act as actants in everyday practices due to their assemblage with the diagnosis of schizophrenia, mediating the bodily, psychological, and social domains of these individuals. This complexity contrasts with the simplification inherent in the predominant biomedical perspective guiding treatment in Chile, which conceptualizes schizophrenia as a chronic condition of biological origin whose primary treatment should be pharmacological.^25^^,^^68^

The results indicate the persistence of a practice in which a diagnosis of schizophrenia implies lifelong treatment with neuroleptics, without the possibility of discharge or cure. This practice, embedded in healthcare dispositifs that promote continuous adherence to medication, has been documented as part of a power dynamic that positions individuals as patients in a relationship of dependency with the psychiatric system^25^. Previous ethnographic research shows how this framework produces an internalization of the status of “chronic mental patient,” affecting self-perception, agency, and the possibility of recovery.^32^

Although medications are valued for alleviating symptoms during acute phases ,^21^ their use is associated with numerous side effects that significantly affect daily functioning, including drowsiness, gastrointestinal problems, motor effects, affective flattening, and metabolic disturbances.^20^^,^^22^^,^^69^ These effects were experienced by participants in this study, demonstrating how they hinder the ability to maintain a work routine, pursue education, or sustain social relationships. The literature consistently shows that the burden of adverse effects compromises adherence to long-term treatment,^23^^,^^37^ and some studies report that a significant proportion of patients discontinue treatment for this reason.^22^

This tension between the perceived need for medication and its side effects has been conceptualized as a “no-exit labyrinth”,^21^ in which individuals develop strategies to manage negative effects, such as adjusting dosages, modifying schedules, or even interrupting treatment-often without medical approval. Such strategies were also identified in this study, revealing individuals’ agency in the face of the mandate of adherence and their efforts to balance crisis prevention with the pursuit of a livable life.

The findings also show that medications can operate as actants in the production of stigma, as they become visible markers and generate observable effects that render individuals, in Goffman’s^47^^)^ terms, discreditable.

In social and labour contexts, the visible effects of pharmacological treatment (such as tremors, salivation, and drowsiness, among others) become salient attributes for others, exposing individuals to discrediting. At the same time, medication itself becomes a marker that makes it difficult to conceal the diagnosis, as pharmacological treatment is often required for employment and monitored within workplace inclusion processes. This finding is consistent with multiple studies showing that psychotropic medications not only impact physical and emotional health but also contribute to self-stigma and social rejection.^23^^,^^38^ As has been noted, the stigma associated with the use of antipsychotic medication seriously undermines access to employment and the maintenance of social relationships.^15^^,^^26^

In line with critiques emerging from psychiatric survivor movements and proposals grounded in the recovery model, these findings call into question the hegemony of treatments centered exclusively on pharmacotherapy, as they fail to consider the multiple implications of their everyday use.

This issue becomes even more significant in the field of work and workplace inclusion. Although Chile has made normative progress toward a rights-based approach for persons with disabilities, as established by Law 21.015, in practice, labour inclusion dispositifs for this population continue to operate under a logic of continuity of pharmacological medical treatment, subordinating workplace supports to medical criteria of stabilization. This reproduces the medicalization of work and restricts individuals’ autonomy.

Finally, the findings challenge the assumptions of the dominant literature that associates therapeutic success with adherence to neuroleptics and participation in the labour market.^8^^,^^11^ While some studies suggest that psychotropic medication may facilitate workplace inclusion, other research indicates that there is no clear correlation between symptom relief and subjective well-being,^20^ and that stigma and social factors play a decisive role in shaping opportunities for employment.^14^ Indeed, recent studies^28^^,^^34^ have shown that individuals who discontinue medication after the acute phase may achieve better functional outcomes and experience fewer hospitalizations.

This study emphasizes that neuroleptics occupy a key position as actants in the experiences of these individuals-a complex and tension-filled position. Although they are experienced as alleviating distress, they also affect bodily, psychological, and social experiences, and operate as markers that reveal the diagnosis and expose individuals to stigma, thereby generating significant barriers to workplace inclusion.

These findings underscore the need to advance toward care strategies that not only consider pharmacological stabilization but also take into account the subjective experiences and social contexts in which working people diagnosed with schizophrenia attempt to build their lives.

## Conclusions

The conclusions of this study make it possible to address the question regarding the role of neuroleptics in the experiences of working people diagnosed with schizophrenia in relation to workplace inclusion. The findings show that neuroleptics are relevant actants in these individuals’ experiences: they are valued for their effect in reducing symptoms, yet their prolonged use entails significant tensions, including physical, emotional, and social side effects that hinder everyday life, expose individuals to stigma, and compromise their opportunities for labour market participation. 

This study contributes to the field by making visible the subjective experiences associated with the use of psychotropic medication, which are often absent from research focused on clinical efficacy. In addition, it problematizes the hegemony of the biomedical approach in workplace inclusion programs, where pharmacological treatment operates as an exclusionary requirement for employment. There is a need to advance toward models of care that recognize the voices of service users and enable greater autonomy in the management of their treatments.

Regarding the limitations of this study, and following the criteria of rigor proposed by Guba and Lincoln,^72^ it is acknowledged that the transferability of the findings-that is, their applicability to other contexts-is contingent upon the specific socioeconomic and cultural conditions of the participants.

Future research could explore experiences of medication reduction or discontinuation in workplace contexts, as well as forms of inclusion based on non-pharmacological approaches, thereby contributing to the rethinking of mental health and employment policies from a rights-based perspective. It is also suggested that future studies examine the role of socioeconomic factors in shaping experiences with neuroleptic use.
